# Hierarchical Particle Approach for Co-Precipitated Amorphous Solid Dispersions for Use in Preclinical In Vivo Studies

**DOI:** 10.3390/pharmaceutics13071034

**Published:** 2021-07-07

**Authors:** Luke Schenck, Christopher Boyce, Derek Frank, Sampada Koranne, Heidi M. Ferguson, Neil Strotman

**Affiliations:** 1 Process Research & Development, Merck & Co., Inc., Kenilworth, NJ 07033, USA; derek.frank@merck.com (D.F.); neil_strotman@merck.com (N.S.); 2 Discovery Pharmaceutical Sciences, Merck & Co., Inc., Kenilworth, NJ 07033, USA; christopher.boyce2@merck.com (C.B.); heidi.ferguson@merck.com (H.M.F.); 3 Preformulation, Merck & Co., Inc., Kenilworth, NJ 07033, USA; sampada.koranne@merck.com

**Keywords:** amorphous solid dispersion, precipitation, spray drying, hierarchical particles, co-processed API, pharmacokinetics, early-stage toxicity studies

## Abstract

Amorphous solid dispersions (ASD) have become a well-established strategy to improve exposure for compounds with insufficient aqueous solubility. Of methods to generate ASDs, spray drying is a leading route due to its relative simplicity, availability of equipment, and commercial scale capacity. However, the broader industry adoption of spray drying has revealed potential limitations, including the inability to process compounds with low solubility in volatile solvents, inconsistent molecular uniformity of spray dried amorphous dispersions, variable physical properties across batches and scales, and challenges containing potent compounds. In contrast, generating ASDs via co-precipitation to yield co-precipitated amorphous dispersions (cPAD) offers solutions to many of those challenges and has been shown to achieve ASDs comparable to those manufactured via spray drying. This manuscript applies co-precipitation for early safety studies, developing a streamlined process to achieve material suitable for dosing as a suspension in conventional toxicity studies. Development targets involved achieving a rapid, safely contained process for generating ASDs with high recovery yields. Furthermore, a hierarchical particle approach was used to generate composite particles where the cPAD material is incorporated in a matrix of water-soluble excipients to allow for rapid re-dispersibility in the safety study vehicle to achieve a uniform suspension for consistent dosing. Adopting such an approach yielded a co-precipitated amorphous dispersion with comparable stability, thermal properties, and in vivo pharmacokinetics to spray dried amorphous materials of the same composition.

## 1. Introduction

Amorphous solid dispersions (ASDs) have solidified their place as a formulation approach with proven effectiveness to increase the in vivo exposure of compounds with poor aqueous solubility [1,2,3,4,5,6,7]. In an ASD, the amorphous phase of an active pharmaceutical ingredient (API), having higher aqueous solubility than the thermodynamically stable crystalline phase, is stabilized against crystallization by embedding, at the molecular scale, in a polymeric material. This polymer reduces mobility of the amorphous API and prevents crystallization on time scales required for manufacturing, storage, and administration [8]. In addition to ensuring a physically stable amorphous phase, the polymer also influences in vivo behavior, preventing crystallization and maximizing bioavailability [9,10,11,12,13]. In the face of APIs of increasing molecular weight and lipophilicity [14], there is significant interest in amorphous drug delivery systems in the pharmaceutical industry. To this aim, a number of manufacturing routes have been explored to generate ASDs, including hot melt extrusion, spray drying, stabilization in mesoporous excipient matrices [15], electrospraying [16], precipitation, and fusion [17] based approaches. The most common of these technologies are hot melt extrusion and spray drying, but, given the high processing temperatures required during hot melt extrusion, spray drying has emerged as a versatile and prevalent route to generate ASDs for early scale through commercial process deployment [18,19,20,21,22,23,24,25].

Despite this preference, spray drying is not without its technical challenges. Small scale techniques to generate Spray Dried Dispersions (SDD) often fail to translate to larger clinical or commercial scale equipment [26], and subtle changes in spray drying conditions can have complex implications for final SDD properties. Ramifications of these process sensitivities include compositional gradients in the final ASD product [22,27,28,29,30,31] which can yield advantages to powder properties or dissolution rate [32,33] but also may come with the risk of generating amorphous materials with reduced physical stability against crystallization. Additionally, the ability to achieve robust, commercial tablet compression operations can be challenging, with subtle changes in processing conditions yielding variable SDD morphology and compressibility [34]. Furthermore, modifications to the ASD composition can affect elastic relaxation and tablet capping propensity [35], with SDD showing high elastic recovery [36]. By comparison, other routes to generate ASDs have been shown to have improved compression performance versus SDD [37]. Spray drying also requires a highly volatile solvent to dissolve API at sufficiently high concentration to yield volumetrically productive commercial processing and represents appreciable additional cost due to capital costs associated with equipment and infrastructure. Despite its widespread use in the pharmaceutical industry, there are clear incentives to broaden the spectrum of available technologies to produce amorphous solid dispersions for use during all stages of drug development and at commercial scale.

An alternative approach to generate amorphous solid dispersions is precipitation. Precipitation is performed by dissolving API and polymer in a common solvent and quickly mixing with a common antisolvent to generate a co-precipitated amorphous dispersion (cPAD). Precipitation was one of the earliest approaches to generate amorphous solid dispersions [38,39] and, alongside its use for small scale development [40], has been demonstrated as a viable alternative to spray drying and HME [41,42,43] while also providing potential opportunistic cost advantages compared to spray drying [44]. Precipitation also fills an important niche for compounds with thermal sensitivity and low solubility in volatile organic solvents, which are constraints to successfully generate ASDs at commercial scale using other processing technologies [45]. A large body of precipitation-based routes to achieve ASDs has focused on precipitating ionic polymers using acidified aqueous antisolvents. However, recent work has expanded the scope of precipitation to include fully organic solvent/antisolvent systems with application to non-ionic polymers and surfactants [46].

One intrinsic advantage of a precipitation-based route is the ability to precisely characterize the process governing phase transition using model systems [47,48]. Rotor stator devices offer distributive and dispersive mixing required to rapidly incorporate a viscous stream with a non-viscous stream [49]. They are low cost and highly scalable [50]. This approach has an added benefit when exploring feasibility studies with limited quantities of API, where the cPAD material can be generated from a solvent and anti-solvent system in situ to directly form the dosing vehicle. A class 3 solvent, such as DMSO, pairs nicely with a viscosity modifying anti-solvent, wherein the final uniform suspension can be dosed directly for early pharmacokinetic studies (Supplemental Information).

Larger doses and later studies can necessitate isolation of the cPAD material from the suspension. Prior studies have shown that, while the physical properties of isolated cPAD material can present challenges, this can be overcome with a hierarchical particle approach [51]. In particular, although cPAD material can be generated and isolated at the batch scale, low bulk density and high surface area can lead to poor wettability. While wettability may or may not impact in vivo performance, it does challenge conventional workflows for in vitro assessment of ASD properties such as dissolution rate [51]. Additionally, for toxicity studies, the ability to generate a uniform suspension, achieve high max feasible doses with the suspension, and uniformly administer the suspension is paramount. 

In the hierarchical particle approach, cPAD material is dispersed in a soluble excipient matrix. Although similar approaches have been applied to SDD [52], the procedure to generate cPAD material is easily coupled with a step to form these hierarchical particles. A robust strategy to prepare co-precipitated amorphous material with favorable properties at the bench scale is crucial to utilize this material in conventional workflows for solid form screening and toxicity testing in drug development. Many comparative studies of ASDs assess bioperformance of either dried powders or formulated tablets. This manuscript instead investigates the performance of SDD and cPAD materials as suspensions in a common vehicle for toxicity studies. Such slurry formulations are critical for early preclinical studies to understand pharmacokinetic behavior and establish safety margins prior to clinical administration. Although spray drying can be used to deliver amorphous materials for these purposes, because the spray drying conditions and SDD material properties do not translate from the bench scale, it is more resource-efficient to develop a co-precipitation route which can enables a wide range of delivery scales. 

This work details a generalizable procedure to prepare ASDs via co-precipitation with controllable properties that can be readily applied in toxicity studies and in downstream in vitro assays to characterize and predict the performance of amorphous materials. Pharmacokinetic studies were conducted to assess the bioperformance of cPAD relative to SDD, making the case for broader industry adoption of co-precipitation to generate amorphous materials. The efforts also afford a comparison of the ASD formation process on resulting PK, a topic having limited evaluation or prior discussion in the literature.

## 2. Materials and Methods

### 2.1. Materials

**Figure 1:**
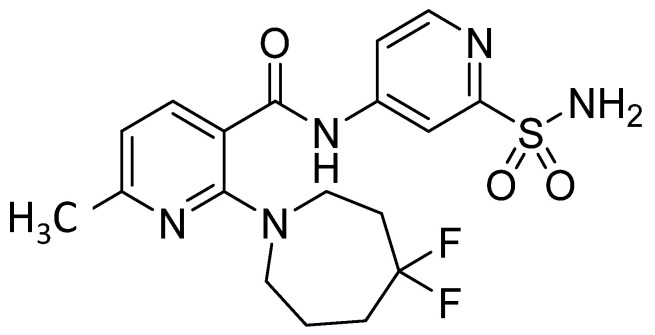
Structure of compound A.

Compound A (Figure 1) was synthesized by Merck & Co. Inc., Kenilworth, NJ, USA. The polymer HPMCAS, L grade, was purchased from ShinEtsu. Lactose was purchased from Foremost and micronized using a 2-inch Micron-Master spiral jet mill (Jet Pulverizer, Moorestown, NJ, USA) operating with nitrogen pressures at 125 PSI for the injector and 65 PSI for the grinder. These conditions resulted in lactose having an × 50 of approximately 1.8 microns as measured using static light scattering system with a dry disperser at 3.5 bar pressure (Sympatec, Pulverhaus, Germany). Micronized lactose was used to allow for the most efficient surface coverage during coating on the cPAD. Vitamin E TPGS was purchased from BASF. 

### 2.2. cPAD Preparation

Compound A and HPMCAS-L were co-dissolved at 40 and 80 mg/mL, respectively, in THF. The antisolvent, *n*-heptane, was pre-cooled to −8 °C. While the heptane was recirculating through a Quadro HV0 rotor-stator mill operating at a rotor speed of 70 m/s, the THF stream was added via peristaltic pump at a 1 to 10 volumetric ratio of solvent to antisolvent.

Additional water-soluble excipients were added post precipitation, with these excipients being insoluble in the final THF/*n*-heptane solvent system. Lactose was added to the final suspension at a concentration of 5.4 mg/mL by charging micronized solids into the mother liquor suspension. Vitamin E TPGS was also added to the suspension to serve as a binder during the evaporative isolation process to the final suspension at a concentration of 1.8 mg/mL. This suspension was then evaporated in a RotoVap (Buchi, New Castle, DE, USA) with a bath temperature of 50 °C, ramping vacuum down to 20 mmHg as quickly as possible without bumping the batch into the distillate receiver. Post evaporative isolation, this approach yielded a hierarchical particle composed of core ASD particles at 33 wt% API, and final hierarchical particle at 20 wt% API. Once fully dried, the hierarchical particle is a dry powder which can recovered from the round-bottom flask.

### 2.3. Spray Drying of COMPOUND A with HPMCAS-L

A spray dried formulation with HPMCAS-L was generated as benchmark formulation. Compound A was dissolved in acetone at 20 mg/mL together with 40 mg/mL HPMCAS-L. The solution was spray dried in a lab scale spray dryer (ProCepT R&D Spray Dryer, Zele, Belgium) with 0.60 mm bifluid nozzle under the following conditions: Inlet air temperature 81 °C, and outlet air temperature 53 °C. The gas flow is 0.4 m^3^/min, and the atomization air rate 5.0 L/min. Solution flow rate is 5 mL/min, and the cyclone pressure is 30 mBar. The spray dried dispersion was secondary dried at 40 °C overnight under vacuum before further use. The material was confirmed to be single phase amorphous solid dispersion by powder x-ray diffraction (Figure S1a) and modulated differential scanning calorimetry (Figure S1b).

### 2.4. Scanning Electron Microscope (SEM)

SEM imaging of the sample was performed using Hitachi SU5000 instrument. Each sample was fixed on aluminum stubs by conductive double-sided carbon adhesive tape and coated with platinum prior to SEM imaging. Images were acquired an accelerating voltage of 2 kV and scanning was conducted with secondary electron detection.

### 2.5. Differential Scanning Calorimetry (DSC)

A differential scanning calorimeter (Discovery DSC, TA Instruments, New Castle, DE, USA) equipped with a refrigerated cooling accessory was used. Dry nitrogen gas was purged at 50 mL/min. Approximately 3–5 mg of sample was weighed in T-zero aluminum pan with pin hole. Sample was equilibrated at 0 °C, heated from 0 °C to 150 °C at 10 °C/min, held for 2 min, cooled back to 0 °C at 10 °C/min and reheated to 200 °C at 10 °C/min.

### 2.6. X-ray Powder Diffractometry (XRPD)

An X-ray diffractometer (D8 Advance; Bruker AXS, Madison, WI, USA) equipped with Si strip one-dimensional detector (LynxEye; Bruker AXS, Madison, WI, USA) was used. The powder samples were exposed to Cu Kα radiation (1.54 Å; 40 kV × 40 mA) over an angular range of 2−40° 2θ with a step size of 0.0196° and a dwell time of 1 s. Data were analyzed using commercially available software (X’Pert HighScore Plus version 2.2 e).

### 2.7. Preparation of Formulations for Oral Pharmacokinetic Studies

Hierarchical particles of Compound A prepared by cPAD (40% HPMCAS-L/10% TPGS/30% lactose) were formulated for oral PK studies by suspending in 0.5% MC (methylcellulose)-5 mM HCl. The mixture was stirred for 30 min, bath sonicated for 20 min, stirred overnight, and then sonicated for 30 min to provide a visually uniform white homogeneous suspension.

Spray-dried amorphous dispersions of Compound A were formulated by suspending in 0.5% MC-5 mM HCl-1% TPGS. The mixture was stirred for 30 min, bath sonicated for 15 min, and then stirred overnight, to provide a visually uniform white homogeneous suspension.

While TPGS was added to the hierarchical cPAD particles to act as a binder, it was recognized it can act as a surfactant and increase solubility of API’s in the toxicity vehicle. As such, Vitamin E TPGS was added to the vehicle for the SDD in order to ensure a fair comparison of the cPAD and SDD PK results.

### 2.8. Ultra-Performance Liquid Chromatography (UPLC)

A quantificatoin of the samples was performed with a Waters Acquity UPLC System (Waters, Milford, MA, USA) with a Waters Acquity BEH C18 (100 × 2.1 mm, 1.7 μm particle size) column with a flow rate of 0.31 mL/min and detection at 210 nm and 254 nm. Mobile phase A consisted of water with 0.1% phosphoric acid and mobile phase B consisted of acetonitrile. A gradient of 5 to 90% mobile phase B was run over 5 min with an additional 2 min hold at 90% B. Compound A and ASD samples were prepared by dilution with 70% acetonitrile/water.

### 2.9. Oral Pharmacokinetic Studies

Male Wistar-Han rats were used for oral administration studies. All animal studies were reviewed and approved by the Merck IACUC (Institutional Animal Care and Use Committee, IACUC Number: 2024-601266-APR; start date 1 April 2019). All animals were fasted overnight before dosing, provided water ad libitum, and fed 4 h following drug treatment. The fasted animals were orally given formulations by gavage (*n* = 3) at a dose of 10 or 100 mg/kg with a dose volume of 5 mL/kg. The vehicle for Compound A cPAD material was 0.5% MC (methylcellulose) with 5 mM HCl. The vehicle for Compound A SDD material was 0.5% MC (methylcellulose) with 5 mM HCl and 1% TPGS. Serial blood samples (250 uL whole blood) were collected at 0.25, 0.5, 1, 2, 4, 8, 12, 18, and 24 h post-dose and placed into EDTA-containing tubes and centrifuged (10,000 rpm for 2 min). Plasma was harvested and stored at −70 °C until analysis. Plasma was protein precipitated with acetonitrile containing 0.1% formic acid and an internal standard analog. Supernatants from the protein precipitation were diluted into water with 0.1% formic acid. Calibrators and unknowns were measured by liquid chromatography-mass spectrometry. Bioanalytical standards were accepted with 25% accuracy and precision. Chromatographic separation was performed on a Thermo Transcend LX2 UPLC (Thermo Scientific, Waltham, MA, USA) with a Waters XSELECT HSS T3 XP (50 × 2.1 mm, particle size 2.5 μm) column with a flow rate of 0.75 mL/min. Mobile phase A consisted of acetonitrile containing 0.1% formic acid. Mobile phase B consisted of water containing 0.1% formic acid. Mass spectrometry quantitation was performed on an AB Sciex 5000 (AB Sciex LLC, Framingham, MA, USA).

## 3. Results and Discussion

### 3.1. Neat cPAD Material 

**Figure 2:**
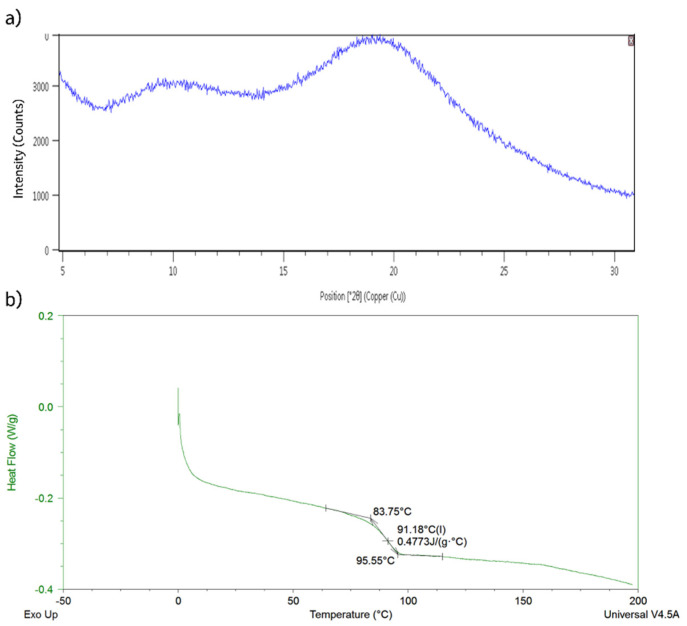
(**a**) PXRD of co-precipitated amorphous dispersion, and (**b**) DSC showing T_g_ of 92 °C for cPAD material.

The generated co-precipitated dispersion was compared to the spray dried intermediate to examine solid-state properties of the resulting material from each formation and isolation route. Shown in Figure 2, the amorphous material formed from precipitation into *n*-heptane is X-ray amorphous and has a T_g_ of roughly 92 °C, which matches the T_g_ of the spray dried intermediate (characterization of the crystalline API is shown in Figure S2).

Despite having similar molecular properties to the SDD, the neat cPAD material was far more difficult to suspend in the tox vehicle in comparison to the SDD. Aggressive reconstitution conditions involving stirring for 10 min followed by sonication for 15 min, and additional stirring for four h at room temperature, even with addition of 0.25 wt% SLS, failed to disrupt aggregates that impact syringability and suspension uniformity. The suspensions at 1 mg/mL and 20 mg/mL concentrations resulted in variable assays, on average at ~80% of the dosing target. By 24 h of mixing, material was better dispersed and doseable, with sampled suspension assay close to the target. However, these difficulties suspending and variable dosing of the cPAD sample were not acceptable in the context of early toxicity screening. These results were similar to previous observations, which could not be fully resolved by adjusting particle size of the cPAD materials [51]. To combat such suboptimal properties, a hierarchical cPAD approach was applied.

### 3.2. Hierarchical cPAD Material

**Figure 3:**
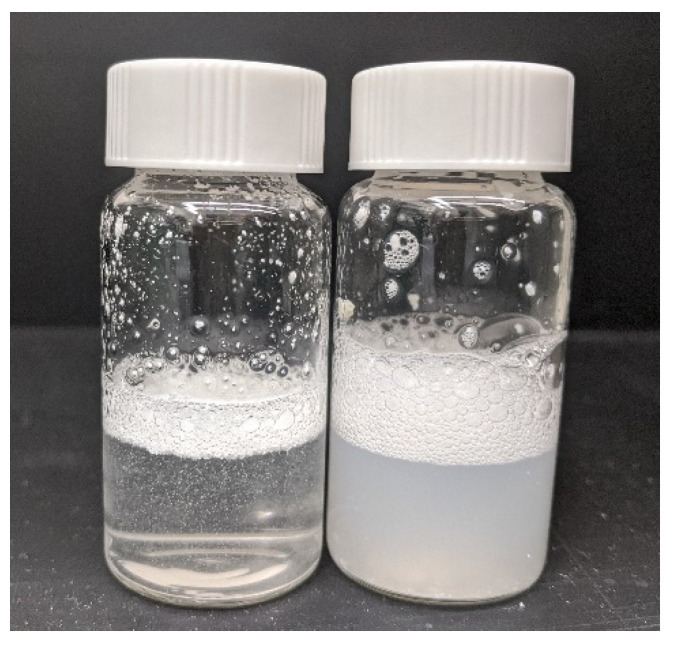
Example comparison between neat co-precipitation amorphous dispersion (left) and hierarchical particle (right) after agitation in DI water.

Following previous work on the use of hierarchical particle design to improve properties of co-precipitated amorphous dispersions [51], a composite particle containing the dispersed amorphous API phase alongside hydrophilic excipients (commonly used excipients include sugars, sugar alcohols, non-ionic polymers, waxes and inorganic salts) was generated to improve the rate of dispersibility and suspension in tox vehicles. Rapid evaporation of solvent thus embeds the amorphous dispersion into a water-soluble matrix, aiding in properties such as dissolution rate, disintegration rate, and bulk powder properties such as flow and compressibility [51]. Leveraging this approach to generate these hierarchical particles overcomes interparticle forces at play in dry powder processing [53], facilitating generation of a more uniform mixture. A sample comparison of re-dispersibility for neat cPAD and a hierarchical particle is shown in Figure 3.

**Figure 4:**
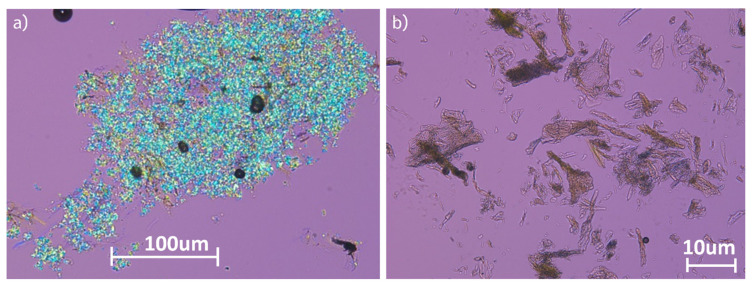
Hierarchical cPAD particle dispersed in (**a**) mineral oil and dispersed in (**b**) aqueous media buffered to a pH of 5.

**Figure 5:**
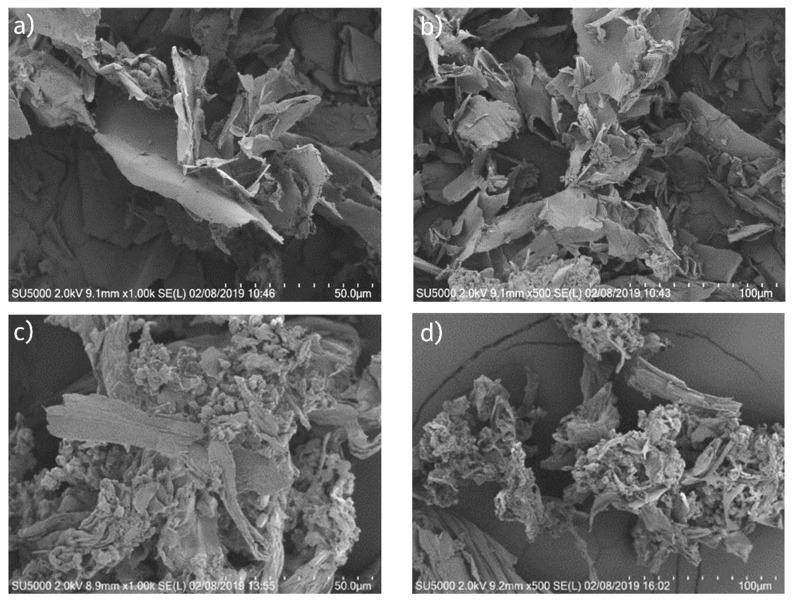
SEM image of cPAD particle isolated without water-soluble excipients, (**a**) 1000× resolution, (**b**) 500× resolution; hierarchical cPAD particle generated by isolating amorphous dispersion with water-soluble lactose matrix, (**c**) 1000× resolution, (**d**) 500× resolution.

The Lactose and TPGS concentrations were titrated up to 30 wt% lactose and 10 wt% TPGS, at which reasonable surface coverage of the cPAD particles was achieved based on empirical observation using polarized light microscopy (PLM). This assessment was assessed by evaluating the isolated solids first suspended in oil, in which none of the hydrophilic components dissolve (Figure 4a), demonstrating cPAD material is embedded in a crystalline excipient. By dispersing the same hierarchical particles in 0.01 N HCl, water soluble excipients dissolve, demonstrating qualitatively that the substrate cPAD particles remain amorphous even when processed with crystalline excipients (Figure 4b). Figure 5a,b shows SEM images of the neat cPAD material with large flakes of high surface area, while Figure 5c,d shows the crystalline hydrophilic excipients coating the surfaces of the hierarchical particles.

HPLC analysis confirmed the composition of the hierarchical particles at 20% drug load. The physical stability against both crystallization and aggregation of the hierarchical cPAD material was compared with the SDD across a series of biorelevant media (including SGF and FaSSIF) and found to be similar through 24 h. In addition, while all suspensions were mixed for four h prior to administration, the hierarchical cPAD particles achieved concentrations at 1 h equivalent to those of SDD achieved after 24 h, suggesting potential for an improved dissolution rate relative to the SDD, see Tables S1 and S2. Wettability and doseability of this hierarchical cPAD is far improved over the neat precipitated dispersion. Similar to measurements in biorelevant media, solubility in tox vehicles was stable against recrystallization over 24 h in MC, acidified MC, and acidified MC containing Vitamin E TPGS. Detailed in Tables S3 and S4, after 18 h in the tox vehicle, ~3 mg/mL of the API had been extracted from the SDD into solution and the remaining is embedded in the amorphous dispersion, indicated by a lack of crystallization by polarized light microscopy. 

**Figure 6:**
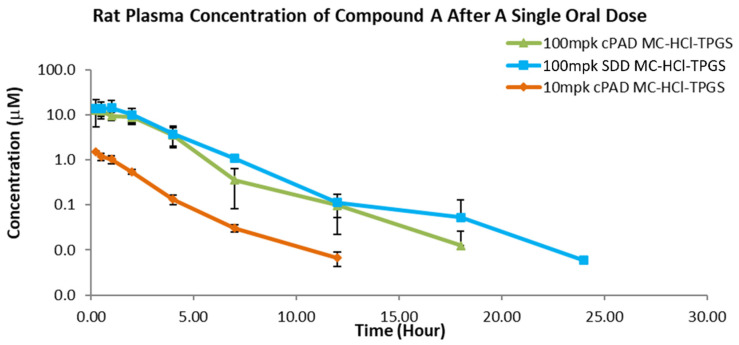
Rat pharmacokinetic profile of drug concentration for cPAD and SDD formulations.

Oral pharmacokinetic studies were performed to compare bioperformance of the hierarchical cPAD and SDD materials, in particular to assess dose linearity of the hierarchical cPAD formulation to demonstrate its potential in toxicity screening. Rat plasma concentration after a single oral dose of Compound A delivered from hierarchical cPAD and SDD was measured over a range of doses to investigate dose proportionality. Plotted in Figure 6, dose proportionality was demonstrated at 10 and 100 mpk dosing for the cPAD material. At 100 mpk, where one could expect to see more differentiation in the limit of poor dispersibility or dissolution of the API formulation, the SDD and cPAD materials showed equivalent concentrations in plasma (Table 1). These results solidify the applicability of hierarchical cPAD formulations in early toxicity studies. 

Demonstrating translatability between processing routes for amorphous solid dispersions remains a goal in the pharmaceutical sciences. Despite the clear differences in morphology between SDD and cPAD of Compound A, we find comparable in vivo exposure. That said, pre-dispersing the amorphous phase in acidified buffer has the effect of extracting API to its amorphous solubility and the rapid dissolution and absorption of the remaining Compound A in the 100 mpk is not resolved by a liberation phase in the pharmacokinetic profile. The quick release of Compound A is likely due to its high solubility in acidic media. A hierarchical particle approach mitigates poor disintegration of the neat cPAD in vitro. Although a significant number of studies have assessed advantages of different processing technologies to prepare amorphous solid dispersions at the bench and batch scales using in vitro characterization methods [54], few studies have directly compared the in vivo pharmacokinetics of amorphous solid dispersions prepared by different formation routes. Factors including particle size/surface area and molecular homogeneity of dispersions afforded by different manufacturing processes for ASDs can impact properties such as physical stability and dissolution rate [41,55,56,57,58]. Few of these have carried forward material to generate in vivo data. Yet, such studies are necessary to assess the risk of altering drug product formulation [59] and to optimize the bioperformance of ASDs and are summarized here [5]. Chiang et al. investigated different methods to prepare amorphous solid dispersions of griseofulvin in HPMCAS [60]. Although dispersions prepared by spray drying, solvent evaporation, and lyophilization showed differential physical stability under accelerated stability conditions, no difference in in vitro dissolution rate or in vivo exposure was observed for unaged samples. Zhang et al. compared amorphous dispersions prepared by hot melt extrusion and spray drying [61]. In this study, particle size differences between each amorphous dispersion resulted in discriminated in vitro dissolution behavior, however in vivo pharmacokinetic profiles were found to be quite similar. Mann et al. compared ASDs prepared by spray drying and co-precipitation [46]. Here, greater tablet strength for co-precipitated ASDs over those formed by spray drying resulted in a longer disintegration time. This difference was reflected in canine pharmacokinetic data, where the cPAD formulation has a greater t_1/2_ and t_max_. Nonetheless, critical pharmacokinetic parameters such as AUC were equivalent for the two systems.

The above data comparing spray dried and co-precipitated dispersions also suggests comparability between each formulation in vivo and, more significantly, implies viability of replacing spray drying with co-precipitation to generate amorphous solid dispersions for small scale toxicity studies. Both spray drying and precipitation have unique applications across the development horizon for a pharmaceutical material. Co-precipitation is a continuous unit operation that generates material agnostic of scale, yet suffers from current regulatory uncertainties [62]. Spray drying has a better developed supply chain despite challenges translating at-scale procedures between processing facilities. The above data demonstrate how a hierarchical particle approach can enable co-precipitated amorphous dispersions for quick deployment in toxicity studies. Potential liabilities of the co-precipitated dispersion, such as high surface area limiting re-dispersibility, can be addressed using a hierarchical particle approach. The equivalent bioperformance of the cPAD and SDD formulations highlights how early toxicity screening can be performed without developing spray drying procedures and instead by leveraging anti-solvent precipitation.

## 4. Conclusions and Next Steps

Both spray drying and co-precipitation are suitable formulation approaches to achieve equal dose linearity for an early-stage compound at Merck Research Laboratories. Formulation development for the purposes of toxicity studies requires a combination of robustness, speed, and generalizability across compounds. Given the many benefits to generating amorphous dispersions by precipitation, it is necessary to develop translatable workflows to mitigate challenges in co-precipitated dispersions. We demonstrate how a hierarchical particle approach can improve dispersibility of cPAD particles to streamline tox vehicle generation for early toxicity studies. Future work will continue to investigate how best to position early-stage formulation workflows to successfully achieve high available concentrations of API, as without a nuanced understanding of how to engineer desirable particle properties, it is possible that poorly soluble API will be eliminated for inadequate in vitro properties—a worrisome prospect in the case of potentially potent and lifesaving in vivo efficacy.

## Figures and Tables

**Table 1:** Summary of pharmacokinetic profiles for hierarchical cPAD and SDD.

Dose (mpk)	Batch	C_max_ Plasma (uM)	C_max_ Std Dev	AUC_0–24_ Plasma (uM*h)	AUC Std Dev
100	SDD	15.7	5.5	45.6	10.6
10	hierarchical cPAD	1.5	0.1	2.7	0.3
100	hierarchical cPAD	12.9	2.6	35.8	4.9

## Data Availability

All data are available upon request.

## Supplementary Materials

The following are available online at https://www.mdpi.com/article/10.3390/pharmaceutics13071034/s1, Figure S1: Characterization of anhydrous, free-base crystalline phase of Compound A by (a) DSC, and (b) PXRD. Figure S2: DSC showing T_g_ of 92 °C for SDD material. Figure S3: DSC showing melt of lactose in hierarchical cPAD at 213 °C, indicating it is the anhydrous phase (melting point depressed due to presence of other components to hierarchical particle). Table S1: Solubility of amorphous solid dispersions and crystalline API in simulated gastric fluid. Table S2: Solubility of amorphous solid dispersions and crystalline API in simulated fasted intestinal fluid (FaSSIF from SIF powder). Table S3: Solubility of spray dried dispersion of Compound A (33% in HPMCAS-L) in the administered dosing vehicle (0.5%MC-5 mMHCl-1% TPGS). The concentration % target is a measure of the concentration of API from a slurry formulation. The solubility value represents the measured solubility in the formulation vehicle by HPLC after filtration of residual solids (an aliquot of formulation was centrifuged at 13,000 rpm in centrifuge tube with Durapore PVDF 0.45 µm Ultrafree filter at 25 °C). Table S4: Solubility of hierarchical cPAD dispersion of Compound A (40% HPMCAS-L/10% TPGS/30% lactose) in the administered dosing vehicle (0.5%MC-5 mM HCl). Figure S4: Polarized light microscopy of 20 mg/mL SDI stored in 0.5%MC-5 mM HCl-1%TPGS after 18 h. Figure S5: Polarized light microscopy of cPAD stored in MC solution after 24 h. Figure S6: PXRD of cPAD after 24h in SGF (red), 24h in MC (blue), 24h in FaSSIF (green). Reference is hemi-hydrate of API (black).
