# Supplementary material for: Hierarchical Particle Approach for Co-Precipitated Amorphous Solid Dispersions for Use in Preclinical In Vivo Studies
Source: Pharmaceutics. 2021 Jul 7;13(7):1034. doi: 10.3390/pharmaceutics13071034 (PMC8308979; doi:10.3390/pharmaceutics13071034)
Supplement: Supplementary file 1 [file pharmaceutics-13-01034-s001.zip › pharmaceutics-1252588-supplementary.pdf]

pharmaceutics

MDPI

Supplementary Materials: Hierarchical Particle Approach for Co-Precipitated Amorphous Solid Dispersions for Use in Preclinical In Vivo Studies

Luke Schenck*, Christopher Boyce, Derek Frank, Sampada Koranne, Heidi M. Ferguson, Neil Strotman

Characterization of API and ASD phases

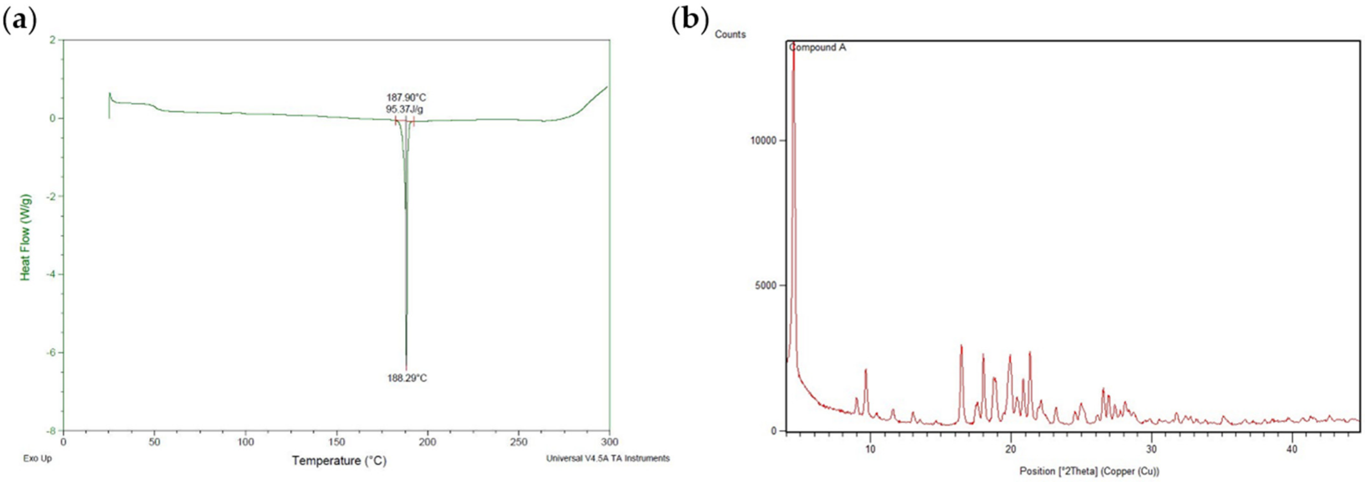

Figure S1. Characterization of anhydrous, free-base crystalline phase of Compound A by (**a**) DSC, and (**b**) PXRD.

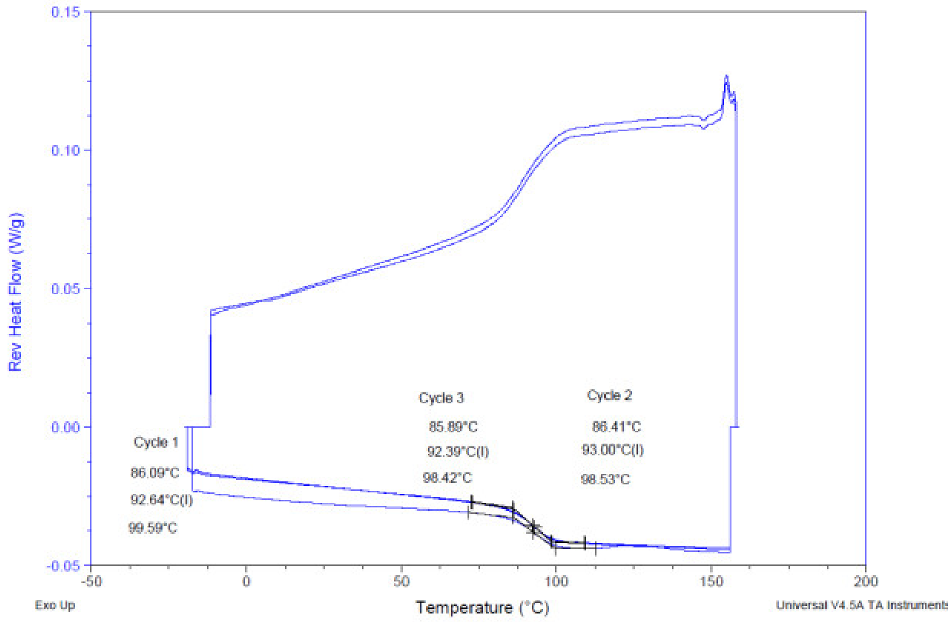

Figure S2. DSC showing T_g of 92 °C for SDD material.

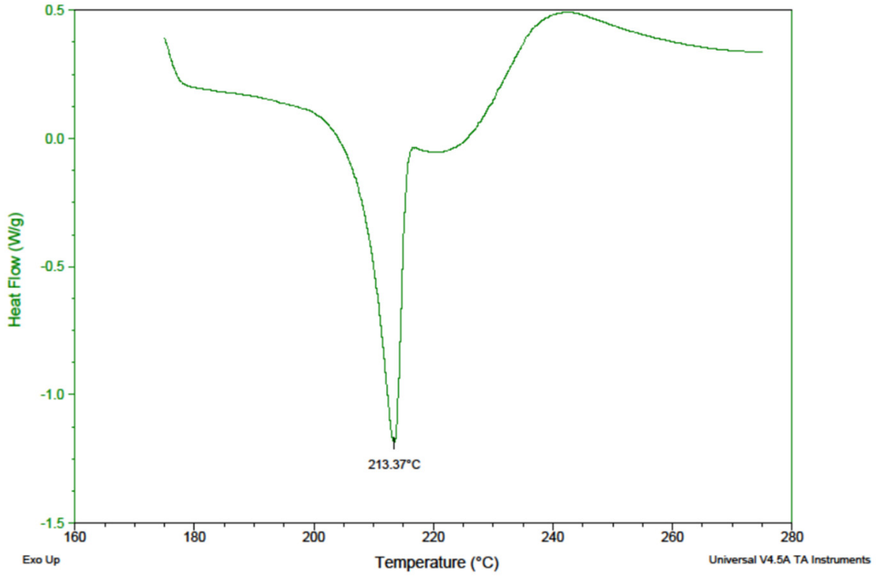

Figure S3. DSC showing melt of lactose in hierarchical cPAD at 213 °C, indicating it is the anhydrous phase (melting point depressed due to presence of other components to hierarchical particle).

Table S1. Solubility of amorphous solid dispersions and crystalline API in simulated gastric fluid.

Material	Solubility (mg/mL) in Simulated Gastric Fluid (SGF)		
	**1 h**	**4 h**	**24 h**
SDD	2.10	2.45	3.31
Hierarchical cPAD	3.11	3.11	3.35
Crystalline API		1.11	1.16

Table S2. Solubility of amorphous solid dispersions and crystalline API in simulated fasted intestinal fluid (FaSSIF from SIF powder).

Material	Solubility (mg/mL) in Simulated Fasted Intestinal Fluid (FaSSIF)		
	**1 h**	**4 h**	**24 h**
SDD	0.44	0.56	0.27
Hierarchical cPAD	1.30	1.18	1.06
Crystalline API		0.03	0.03

Table S3. Solubility of spray dried dispersion of Compound A (33% in HPMCAS-L) in the administered dosing vehicle (0.5%MC-5mMHCl-1% TPGS). The concentration % target is a measure of the concentration of API from a slurry formulation. The solubility value represents the measured solubility in the formulation vehicle by HPLC after filtration of residual solids (an aliquot of formulation was centrifuged at 13,000 rpm in centrifuge tube with Durapore PVDF 0.45 µm Ultrafree filter at 25 °C).

Vehicle	Target API Conc (mg/mL)	Time (h)	Bifringent by PLM?	Description	Concentration %Target	Solubility Value (mg/mL)
0.5%MC-5mMHCl-1% TPGS	19.82	1	No	well dispersed white suspension	129%	2.64
		18	No	well dispersed white suspension	92%	3.63

Table S4. Solubility of hierarchical cPAD dispersion of Compound A (40% HPMCAS-L/10% TPGS/30% lactose) in the administered dosing vehicle (0.5%MC-5mM HCl).

Vehicle	Target API Conc (mg/mL)	Time (h)	Description	Concentration %Target	Solubility Value (mg/mL)
0.5%MC-5mMHCl-1% TPGS	1.3	1	well dispersed white suspension	90%	> 1.3
		24	well dispersed white suspension	107%	> 1.3
	20.0	1	well dispersed white suspension	89%	
		24	well dispersed white suspension	78%	

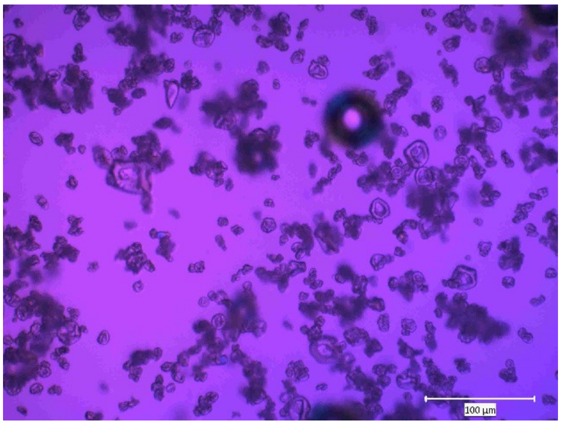

Figure S4. Polarized light microscopy of 20 mg/mL SDI stored in 0.5%MC-5mM HCl-1%TPGS after 18 h.

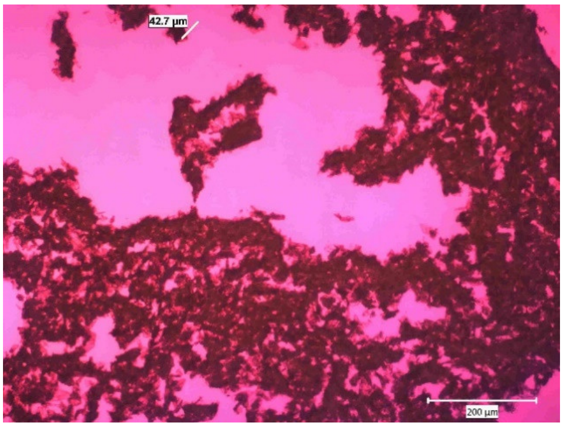

Figure S5. Polarized light microscopy of cPAD stored in MC solution after 24 h.

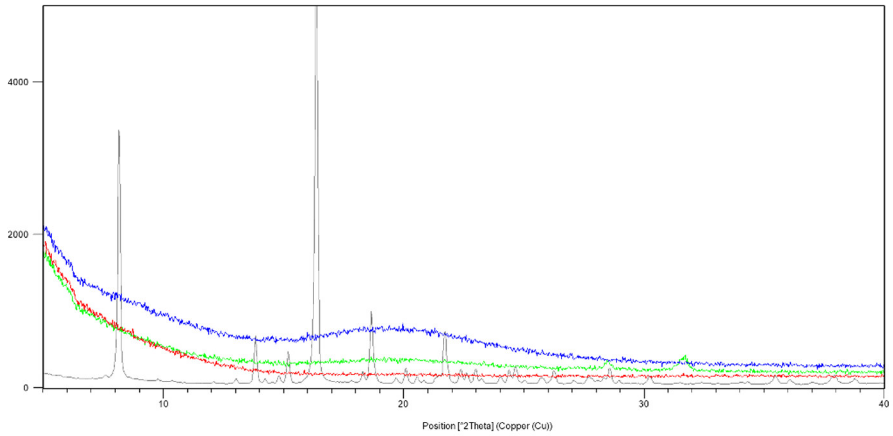

Figure S6. PXRD of cPAD after 24 h in SGF (red), 24 h in MC (blue), 24 h in FaSSIF (green). Reference is hemi-hydrate of API (black).

Representative In Situ cPAD Preparation

API and HPMCAS-M were co-dissolved at 100 and 150 mg/mL, respectively, in dimethylsulfoxide (DMSO). The antisolvent, 0.5% Methocel™/5 mM HCl was pre-cooled in an ice bath. While the antisolvent was mixing with a handheld tissue homogenizer or IKA Ultra Turrax™ Tube Drive set to the highest speed, the DMSO mixture was added dropwise via a syringe and 21G needle at a 1 to 10 volumetric ratio of solvent to antisolvent. The suspension was allowed to mix for 3–5 min until a foamy suspension was generated, with no visible aggregates. The suspension was removed from the ice bath, covered, and stirred at room temperature until foaming reduced (4–18 h) prior to oral administration in rodents. Alternatively, a Thinky Mixer™ can be used to rapidly de-foam suspension prior to administration.
